# Evaluating Cordyceps militaris capsules on post-bronchodilator FEV_1_ decline in patients with COPD: a study protocol for double-blind, randomized, placebo-controlled trial

**DOI:** 10.3389/fphar.2026.1775068

**Published:** 2026-05-25

**Authors:** Siqi Lin, Tianming Liao, Feiting Fan, Yuanbin Chen, Yue Yan, Hangming Dong, Shaofeng Zhan, Yuanbing Zhang, Kun Xiao, Lin Lin, Lei Wu

**Affiliations:** 1 State Key Laboratory of Traditional Chinese Medicine Syndrome, The Second Clinical College of Guangzhou University of Chinese Medicine, Guangdong Provincial Hospital of Chinese Medicine, Guangzhou, China; 2 Guangdong-Hong Kong-Macau Joint Lab on Chinese Medicine and Immune Disease Research, Guangdong Provincial Key Laboratory of Traditional Chinese Medicine for Prevention and Treatment of Refractory Chronic Diseases, Guangzhou, China; 3 China-Japan Friendship Hospital, Beijing, China; 4 Nanfang Hospital Southern Medical University, Guangzhou, China; 5 The First Affiliated Hospital of Guangzhou University of Chinese Medicine, Guangzhou, China; 6 Jiangxi Provincial Hospital of Chinese Medicine, Nanchang, China; 7 The Second People’s Hospital of Baiyun and Guangzhou, Guangzhou, China

**Keywords:** chronic obstructive pulmonary disease, Cordyceps militaris capsules, lung function, post-bronchodilator FEV1, protocol

## Abstract

**Background:**

Chronic Obstructive Pulmonary Disease (COPD) is characterized by a progressive deterioration of lung function, which occurs at its fastest rate in the early stages (GOLD I-II). Preliminary clinical investigation has suggested that *Cordyceps militaris* capsules can improve post-bronchodilator forced expiratory volume in 1 s (FEV_1_) in mild-to-moderate COPD. However, previous studies were limited by the absence of placebo control. Therefore, this trial is designed to evaluate the efficacy and safety of *Cordyceps militaris* capsules versus placebo in improving post-bronchodilator FEV_1_ in patients with mild-to-moderate stable COPD.

**Methods:**

This study is a multi-center, randomized, double-blind, placebo-controlled clinical trial involving 160 eligible patients aged between 40 and 85 years. After a 1-week run-in period, participants are randomized in a 1:1 ratio to receive either *Cordyceps militaris* capsules or placebo capsules for 24 weeks, and are followed for an additional 24 weeks. The primary outcome measure is the change in post-bronchodilator FEV_1_. Secondary outcome measures comprise additional lung function parameters, such as pre-bronchodilator FEV_1_, pre- and post-bronchodilator measures of forced vital capacity (FVC), FEV_1_/FVC ratio, mid-expiratory flow (MMEF), forced expiratory flow 75% (FEF75), forced expiratory flow 50% (FEF50). In addition, frequency of acute exacerbations, time to the first acute exacerbation, COPD Assessment Test (CAT), Cough and Sputum Assessment Questionnaire (CASA-Q), EuroQol five-dimension five-level questionnaire (EQ-5D-5L), and Modified Medical Research Council dyspnea scale (mMRC) are also included.

**Discussion:**

This study is designed to evaluate the therapeutic potential of *Cordyceps militaris* capsules and to provide a traditional Chinese medicine intervention for preserving lung function in mild-to-moderate stable COPD.

**Clinical Trial Registration:**

https://www.chictr.org.cn/, ChiCTR2400086743, identifier, ChiCTR2400086743.

## 1 Introduction

Chronic obstructive pulmonary disease (COPD) is a heterogeneous pulmonary disorder primarily caused by structural abnormalities in the airways and/or alveoli. These pathological changes lead to impaired gas exchange and persistent airflow obstruction, which typically worsen over time and is only partially reversible, thereby distinguishing COPD from other obstructive lung diseases (Global Initiative for Chronic Obstructive Lung Disease, 2025; Chronic Obstructive Pulmonary Disease Group of Chinese Thoracic Society and Chronic Obstructive Pulmonary Disease Committee of Chinese Association of Chest Physicians, 2021). Globally, COPD affected an estimated 212.3 million people, and was responsible for 3.3 million deaths and 74.4 million disability-adjusted life years (Collaborators, 2020; Safiri et al., 2022). Given the aging population and growing number of smokers, the prevalence of COPD is expected to increase further, exacerbating its already substantial social and economic burdens (De Oca et al., 2025). Consequently, the challenges associated with the prevention and control of COPD are becoming increasingly severe.

An accelerated decline in lung function, particularly forced expiratory volume in one second (FEV_1_), is a primary risk factor for chronic respiratory diseases. This decline is most commonly caused by smoking in adulthood (Zhang et al., 2024). The Global Initiative for Chronic Obstructive Lung Disease (GOLD) categorizes the severity of COPD into four grades based on the post-bronchodilator FEV_1_ percent predicted (FEV_1_%pred). Studies have demonstrated that mild-to-moderate COPD (GOLD I-II), accounting for approximately 92.7% of all cases, frequently presents with minimal or mild symptoms (Zhou et al., 2017; Lee et al., 2016). However, this phase is paradoxically characterized by rapid deterioration of lung function, with patients experiencing an annual FEV_1_ decline of 49-60 mL/year (Callejas et al., 2016). By the time overt symptoms such as dyspnea and respiratory distress emerge, patients have typically sustained substantial and often irreversible lung damage (Hurst et al., 2021; Barrecheguren et al., 2018). However, long-term pharmacological intervention can improve respiratory outcomes in patients with mild-to-moderate COPD (Wu et al., 2025).

Current pharmacological interventions for improving lung function in COPD remain limited, with Western medicine predominantly focusing on inhaled therapies. A clinical trial has demonstrated that tiotropium bromide can attenuate the annual rate of FEV_1_ decline by 22 mL/year in mild-to-moderate COPD (Zhou et al., 2017). However, GOLD refrains from recommending any specific pharmacological regimen for mild-to-moderate COPD, citing insufficient clinical evidence (Global Initiative for Chronic Obstructive Lung Disease, 2025). Therefore, there is an urgent need for more rigorous clinical trials to establish evidence-based therapeutic regimens for managing mild-to-moderate COPD.

*Ophiocordyceps sinensis* (Berk.) G.H. Sung et al. (syn. *Cordyceps sinensis*) is a highly valued medicinal fungus in Traditional Chinese Medicine (TCM) (Wang et al., 2016). A meta-analysis has demonstrated that the adjunctive use of *Cordyceps sinensis* prescription with conventional Western medicine can significantly improve lung function parameters, particularly FEV_1_%pred and forced expiratory volume in 1 s/forced vital capacity (FEV_1_/FVC) ratio, in patients with COPD (Yu et al., 2019). At the molecular level, Zhou et al. revealed that *Cordyceps sinensis* regulates glycerophospholipid and sphingolipid metabolism by targeting PLA2G4E and B4GALT4 proteins via the PI3K-AKT pathway. This mechanism may underlie its anti-inflammatory and lung-protective effects against COPD (Zhou et al., 2025). However, the rarity and high cost of wild *Cordyceps sinensis* have driven the development of industrialized fermentation techniques for *Cordyceps militaris* (L.) Fr. 1818 production. This cultivated species contains analogous bioactive compounds and represents a sustainable, clinically viable alternative for medicinal applications (Das et al., 2010).

Modern pharmacological studies have identified multiple bioactive compounds in *Cordyceps militaris*, including cordycepin, polysaccharides, ergosterol, and mannitol, which exhibit various physiological effects such as immunomodulation, antibacterial and anti-inflammatory activity, and antitumor properties (Olatunji et al., 2018). In a multicenter randomized controlled trial, treatment with *Cordyceps militaris* capsules for 24 weeks increased FEV_1_ by 140 mL in mild-to-severe COPD patients, compared to a 10 mL decrease in the Bailing capsule group (Li et al., 2025). Notably, in the mild-to-moderate COPD subgroup, *Cordyceps militaris* capsules improved post-bronchodilator FEV_1_ by 86 mL versus a 41 mL decline in the control group (*P* < 0.05). Although the study lacked placebo control and used acute exacerbation events as the primary endpoint, the findings remain promising.

Therefore, this study is designed as a multi-center, double-blind, placebo-controlled randomized trial to evaluate the efficacy of *Cordyceps militaris* capsules versus placebo in improving post-bronchodilator FEV_1_ in mild-to-moderate COPD, and will provide evidence for a TCM intervention for preserving lung function in patients with mild-to-moderate COPD.

## 2 Methods and analysis

### 2.1 Investigational medications

All investigational medications, including both the *Cordyceps militaris* capsules and placebo capsules, have been manufactured by Jilin Zhongsheng Pharmaceutical Co., Ltd.

*Cordyceps militaris* capsules (National Drug Approval Number: Z20030035) are the cultured form of *Cordyceps militaris* (L.) Fr. 1818, a species belonging to the genus *Cordyceps* and the family Clavicipitaceae*.* The *Cordyceps militaris* strain is inoculated onto oak silkworm pupae and cultured at 15 °C-22 °C for approximately 45 days. After the stroma has grown, the material is harvested, dried at 60 °C and ground into a fine powder. After the addition of excipients through subsequent processing steps, the final capsules are produced. The detailed preparation of *Cordyceps militaris* capsules is shown in Supplementary Material 1. Jilin Zhongsheng Pharmaceutical Co., Ltd. has conducted constituent analysis and quality control testing through high-performance liquid chromatography and gas chromatography. The voucher specimens have been stored in the company warehouse. The composition analysis of *Cordyceps militaris* capsules and quality-control fingerprints are shown in Supplementary Material 2. In addition, a drug testing report for *Cordyceps militaris* capsules with detailed information is provided in Supplementary Material 3. No significant adverse reactions have been reported in previous clinical studies, suggesting a favorable safety profile (Li et al., 2025).

The main components of the placebo capsules are dextrin, corn starch, caramel, tartrazine, lentinus edodes (shiitake) powder, and dried flatfish powder. The placebo capsules match *Cordyceps militaris* capsules in smell, appearance, taste, and weight, thereby ensuring effective blinding. The preparation is shown in Supplementary Material 1, and drug testing reports are shown in Supplementary Material 4.

### 2.2 Study design

**FIGURE 1:**
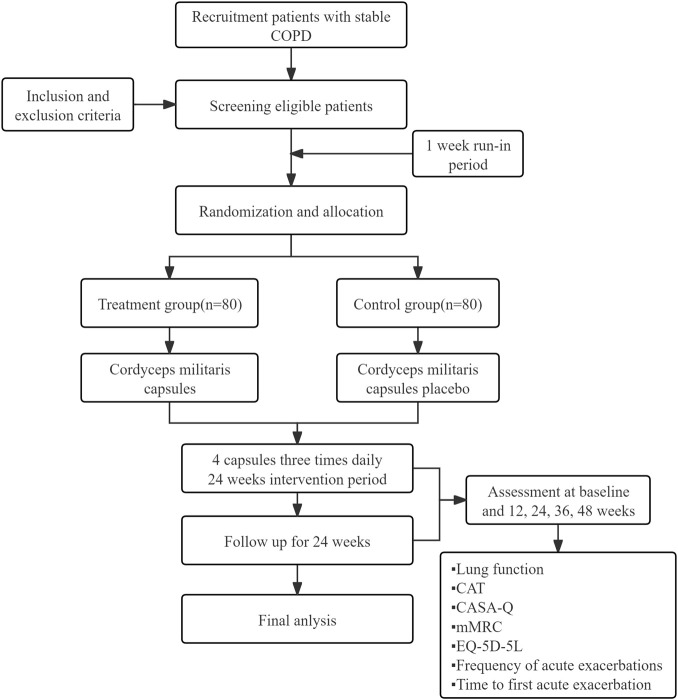
Study flow chart. COPD, Chronic Obstructive Pulmonary Disease; CAT, COPD Assessment Test; CASA-Q, Cough and Sputum Assessment Questionnaire; EQ-5D-5L, EuroQol five-dimension five-level questionnaire; mMRC, Modified Medical Research Council dyspnea scale.

This is a multi-center, randomized, double-blind, parallel-group, placebo-controlled clinical trial. The trial has been initiated by the Guangdong Provincial Hospital of Chinese Medicine. After a 1-week run-in period, a total of 160 eligible participants are randomized to the treatment group (*Cordyceps militaris* capsules) or the control group (placebo capsules) in a 1:1 ratio. Participants receive a 24-week intervention and an additional 24-week follow-up period. Study visits will take place at baseline and weeks 12, 24, 36, and 48 within 30 days of each scheduled visit. Participants are required to provide written informed consent. The protocol follows the principles of the Standard Protocol Items: Recommendations for Interventional Trials (SPIRIT) Guidelines. A flowchart of the trial is shown in Figure 1. The schedule of enrollment, interventions, and assessments is presented in Table 1.

**TABLE 1:** A standard protocol items: recommendations for interventional trials (SPIRIT).

Trial period						
Timepoint	Enrollment		Post randomization			Close out
	-t_1_-0 −1 week	0 0 weeks	t_1_ 12 weeks	t_2_ 24 weeks	t_3_ 36 weeks	t_4_ 48 weeks
Enrollment						
Eligibility screen	×	​	​	​	​	​
Informed consent	×	​	​	​	​	​
Demographic data	×	​	​	​	​	​
Medical history	×	​	​	​	​	​
Allergic history	×	​	​	​	​	​
Randomization	​	×	​	​	​	​
Intervention or comparator						
Intervention	​	×	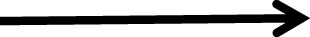	×	​	​
Comparator	​	×	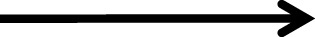	×	​	​
Assessments						
Lung function	(×)	×	×	×	×	×
CAT	​	×	×	×	×	×
CASA-Q	​	×	×	×	×	×
mMRC	​	×	×	×	×	×
EQ-5D-5L	​	×	×	×	×	×
Assessment of acute exacerbation	×	×	×	×	×	×
General physical examination	×	×	×	×	​	​
Blood routine	×	​	×	×	​	​
Liver and kidney function	×	​	×	×	​	​
AEs	​	×	×	×	×	×
Others						
Biological samples	​	×	​	×	​	×
Distribution of drugs	​	×	×	​	​	​
Recycling of drugs	​	​	×	×	​	​
Completion of CRF	×	×	×	×	×	×
Summary of study conclusion	​	​	​	​	​	×

### 2.3 Participants

#### 2.3.1 Recruitment and trial setting

Participants are recruited from multiple hospitals across China. These participating centers include Guangdong Provincial Hospital of Chinese Medicine, the First Affiliated Hospital of Guangzhou University of Chinese Medicine, the Second People’s Hospital of Baiyun and Guangzhou, Nanfang Hospital Southern Medical University, China-Japan Friendship Hospital and Jiangxi Provincial Hospital of Chinese Medicine. Participants are screened and assessed primarily in outpatient clinical settings by researchers. Additionally, recruitment advertisements may also be used to identify eligible patients. Only patients who meet all the inclusion criteria and none of the exclusion criteria will be enrolled. Enrolled participants undergo treatment and follow-up in strict accordance with the study protocol. The study commenced on 9 July 2024. Patient recruitment is expected to be completed by late 2026, and the entire trial is projected to conclude by late 2027.

Detailed baseline characteristics are collected for all enrolled participants, including sex, age, medical history, allergy history, disease duration, concomitant medications, smoking status (current, former, or never smoker), smoking index, and duration of smoking cessation.

#### 2.3.2 Diagnostic criteria of diseases

GOLD 2024 (Global Initiative for Chronic Obstructive Lung Disease, 2024) is used for the diagnosis of COPD, grading of pulmonary function, and disease staging.

1. Western medicine diagnostic criteria for COPD:
① Symptoms: Dyspnea, and/or chronic cough and sputum;
② Risk factors: Smoking, exposure to smoke from cooking and heating fuels, exposure to occupational dust and chemicals, or a family history of COPD;
③ Lung function: Post-bronchodilator FEV_1_/FVC <70%;
④ Exclusion: Other airflow restricted diseases causing similar symptoms, such as asthma, bronchiectasis.
2. Grading of lung function

GOLD I: Post-bronchodilator FEV_1_/FVC <70% and post-bronchodilator FEV_1_%pred ≥80%.

GOLD II: Post-bronchodilator FEV_1_/FVC <70% and 50% ≤ post-bronchodilator FEV_1_%pred <80%.

3. Staging of COPD

Stable stage: Cough, sputum, and dyspnea are stable or mild, and the patient has returned to baseline status prior to acute exacerbation.

Acute exacerbation: An event characterized by worsening dyspnea and/or worsening cough and sputum over a period of less than 14 days (a sudden deterioration of respiratory symptoms beyond the usual variation) (Global Initiative for Chronic Obstructive Lung Disease, 2024). The cardinal symptom is worsening dyspnea, typically accompanied by wheezing, chest tightness, increased cough frequency, increased sputum volume, altered sputum characteristics (e.g., color and/or viscosity), and fever.

4. Acute exacerbation severity is classified as:

Mild: Treated with short-acting bronchodilators only.

Moderate: Treated with short-acting bronchodilators and antibiotics, with or without oral glucocorticoids.

Severe: requiring hospitalization, emergency department treatment or intensive care unit admission.

#### 2.3.3 Diagnostic criteria of syndrome of lung qi deficiency

Lung qi deficiency syndrome is diagnosed according to Guidelines of integrated Chinese and western medicine for diagnosis and treatment of chronic obstructive pulmonary disease (2022) (Li et al., 2023).

1. Cough or wheezing, shortness of breath, which aggravated while moving;
2. Mental fatigue, lack of strength, or spontaneous sweating;
3. Aversion to wind, easy to catch cold;
4. Light tongue with white coating.

The syndrome can be diagnosed when meeting three items of the above ①–④.

#### 2.3.4 Inclusion criteria

Participants who meet the following criteria are included:

1. Aged 40-85 years;
2. Diagnosed with COPD according to the criteria of the GOLD 2024 and Guidelines for the Diagnosis and Management of Chronic Obstructive Pulmonary Disease (Revised Version 2021), with lung function classified as GOLD I-II;
3. Stable COPD, with no respiratory infections or acute exacerbations of COPD within 4 weeks before screening;
4. With the TCM syndrome of lung qi deficiency;
5. Informed and signed the informed consent form.

#### 2.3.5 Exclusion criteria

Participants with any of the following conditions are excluded.

1. With bronchiectasis, asthma, malignant lung tumor, active pulmonary tuberculosis etc.,
2. With an acute exacerbation of COPD at screening or within 4 weeks before screening;
3. Inability to cooperate with pulmonary function testing;
4. With long-term use of traditional Chinese medicine diet such as astragalus, ginseng, Codonopsis;
5. With severe cardiovascular, cerebrovascular, liver or kidney diseases, mental disorders;
6. With abnormal liver function [ALT or AST ≥2 times the upper limit of normal (ULN)] or abnormal kidney function (serum creatinine ≥2 times ULN);
7. Patients with a history of allergy or hypersensitivity reaction to the investigational product or its components, or patients believed by the investigator to have diseases that should prohibit the use of the investigational drug;
8. Patients who are participating in or planning to participate in, other clinical trials with 1 month before screening or during the observation period;
9. Deemed ineligible for the study by the investigator.

#### 2.3.6 Withdrawal/termination criteria

Participants are withdrawn from the trial if they meet any of the following criteria.

1. Serious adverse events (SAEs) related to the investigational treatment occur.
2. Serious complications, allergic reactions, clinically significant liver or kidney dysfunction, significant physiological changes that occur during the trial.
3. Continued participation poses unacceptable risks to the participant, as judged by the principal investigator.
4. Participants who experience recurrent infections during treatment will be withdrawn under the automatic withdrawal criteria. Specifically, if a participant experiences two or more acute exacerbations within 1 month requiring emergency department visits or hospitalization, the participant will be automatically withdrawn from the study to mitigate the risk of clinical deterioration and safeguard participant welfare.

### 2.4 Randomization and allocation

The randomization sequence has been generated using a central randomization system. The randomization procedure was programmed using SAS 9.2 software to generate the randomization sequence. In this study, stratified block randomization was designed with airflow limitation severity (GOLD I and GOLD II) as the stratification factor. The researchers use a centralized randomization system to perform participant allocation. Participants are allocated to either the treatment or control group in a 1:1 ratio. The statistical unit maintains centralized control over all study identifiers (center numbers, sequence assignments, randomization codes, and drug numbers) to preserve blinding and prevent bias.

### 2.5 Blinding

This is a double-blind trial, and the blinding and unblinding processes are conducted by independent unblinded personnel in accordance with the standardized double-blind trial procedures. These measures ensure that the investigators, relevant research staff, and participants remain blinded to the treatment assignments. The test drug and placebo are designed to match as closely as possible in terms of appearance, smell, and other physical characteristics. All drug packages are prepared according to double-blind standards, ensuring that no distinguishing features on the outer packaging reveal the treatment groups. An independent unblinded staff member, who is not involved in the trial, is responsible for the drug allocation. A documented record of the blinding process is maintained and securely stored for verification. This trial employs a central randomization system for emergency unblinding rather than paper-based unblinding letters. Emergency unblinding is permitted only in critical situations in which the investigator deems it necessary to take urgent measures to ensure patient safety. In the event of unblinding, the date, time, and justification are promptly documented. Additionally, any associated adverse events must be recorded according to the trial protocols.

### 2.6 Sample size calculation

The sample size has been calculated using PASS software (Version 15.0.5) based on a superiority test design, with assumptions derived from prior subgroup analyses of *Cordyceps militaris* capsules in the treatment of COPD. The primary endpoint is the change in the post-bronchodilator FEV_1_, with an assumed mean difference of 110 mL between groups, and standard deviations set at 250 mL for the treatment group and 201 mL for the control group (Shergis et al., 2019). A one-sided superiority test is conducted with α = 0.025 and Power = 1-β = 0.90, assuming equal allocation between groups. The sample size calculation indicates that 68 participants per group (N = 136) are required to detect the specified treatment effect. To accommodate an anticipated 15% attrition rate, the final sample size is increased to 160 participants (80 per group).

### 2.7 Interventions

1. Treatment group: Participants in the treatment group receive *Cordyceps militaris* capsules [brand name: Ke Cai], produced by Jilin Zhongsheng Pharmaceutical Co., Ltd., for 24 weeks (four capsules per dose, three times daily).
2. Control group: Patients in the control group receive *Cordyceps militaris* placebo capsules, produced by Jilin Zhongsheng Pharmaceutical Co., Ltd., for 24 weeks (four capsules per dose, three times daily).

Under the supervision of their respiratory physicians, participants may continue pre-existing regimens of long-acting anticholinergics or long-acting β_2_-agonists, either as monotherapy or in combination with glucocorticoids. However, other respiratory drugs are prohibited, such as theophylline, corticosteroid monotherapy, antibiotics, mucolytics, and antitussives (Wu et al., 2014). For acute symptom relief (e.g., clinically significant dyspnea or respiratory distress), salbutamol sulfate metered-dose inhalers may be used as needed. Acute exacerbations are managed according to guidelines, including measures such as controlled oxygen therapy, antibiotics, bronchodilators, and systemic corticosteroids. All exacerbations require documentation of temporal characteristics (onset and offset), precipitating factors, and pharmacological interventions. If emergency medications are required within 6 h prior to a scheduled visit, the visit window may be extended to ensure lung function test validity.

In principle, the use of TCM and patent medicines other than the study drug, including any dosage form, is not allowed after enrollment. If an acute exacerbation occurs, the trial drug should be continued without concomitant TCM whenever possible. If TCM is necessary, detailed records must be maintained in the combined medication record form. For patients with comorbidities (such as hypertension, diabetes, etc.), the original regimen for combined medications can be maintained. During the follow-up period, if any TCM or Western medicine is used, detailed records should be kept. All combined medications must be recorded in detail. Unused study medication should be returned.

### 2.8 Outcomes and measurements

The time points of the study data collection for outcome measurements are shown in Table 1.

The primary outcome is change in the post-bronchodilator FEV_1_. Lung function assessments are performed once each at baseline, weeks 12, 24, 36, and 48. Secondary outcomes include additional lung function parameters, such as pre-bronchodilator FEV_1_, pre- and post-bronchodilator measures of FVC, FEV_1_/FVC ratio, mid-expiratory flow (MMEF), forced expiratory flow 75% (FEF75) and forced expiratory flow 50% (FEF50). In addition, frequency of acute exacerbations, time to the first acute exacerbation, COPD Assessment Test (CAT), Cough and Sputum Assessment Questionnaire (CASA-Q), EuroQol five-dimension five-level questionnaire (EQ-5D-5L), and Modified Medical Research Council dyspnea scale (mMRC) are also included. These outcomes are also measured once each at baseline, weeks 12, 24, 36 and 48.

Blood, sputum and stool samples are collected from at least 60 participants enrolled in the Guangzhou center for metagenomics (airway and intestinal flora), transcriptomics, metabolomics, proteomics analyses and induced sputum cell classification. Biological samples are collected at baseline, weeks 24 and 48 after treatment, following standard operating procedures for acquisition, processing, and cryopreservation.

### 2.9 Safety assessments

Safety assessments include the following:

1. Basic vital signs including temperature, heart rate, respiratory rate, and blood pressure are measured once at screening, baseline, weeks 12 and 24.
2. Laboratory tests including routine blood tests, liver function and kidney function tests are performed once at baseline, weeks 12 and 24.
3. Adverse events (AEs) are evaluated and recorded on the case report form (CRF).

### 2.10 Data management

In order to enhance participant retention and ensure complete follow-up, investigators contact participants by telephone to schedule and remind them of follow-up visits. This study utilizes an Electronic Data Capture (EDC) system and paper-based CRFs. The research center must maintain original source documents for all participants. Throughout the study, investigators or their designated clinical research coordinators enter source data into the EDC system in a timely and accurate manner. Following data entry and submission, the system performs automated logic checks. Subsequently, clinical monitors, data managers, and medical reviewers conduct multi-dimensional audits. Discrepancies identified during monitoring are electronically issued as queries to the originating personnel via the EDC system. After comprehensive verification by the principal investigator, statistician, and data manager, the study database will be permanently locked to preserve trial integrity, except under extraordinary circumstances requiring oversight by the ethics committee. The locked database will then be transferred to the statistical analysis team.

### 2.11 Statistical analysis

The analysis will be performed using the intention-to-treat principle on the full analysis set, with additional analyses conducted on the per-protocol set and safety set. Only authorized study personnel will be able to view and manage data. The between-group comparison of acute exacerbation frequency will be conducted using a Poisson regression model that includes treatment group as the independent variable, annualized exacerbation rate as the dependent variable, and stratified randomization factors as adjustment covariates. Least squares means with corresponding 95% confidence intervals (with appropriate transformations when required) will be estimated from the specified model to evaluate between-group differences. Continuous variables will be analyzed using appropriate parametric (two-sample t-test or adjusted t-test) or nonparametric (Wilcoxon rank-sum test) methods, while categorical variables will be evaluated using either Pearson’s chi-square test or Fisher’s exact test. Time-to-event endpoints will be analyzed using Kaplan-Meier methodology, with median event times and interquartile ranges (IQR, 25th-75th percentiles) estimated for each group. Between-group differences will be assessed using log-rank tests. For missing data, the last observation carried forward method will be employed for imputation. For the center effect analysis, continuous variables will be analyzed using analysis of covariance or meta-analysis, while categorical variables will be assessed using Cochran-Mantel-Haenszel statistics.

Baseline demographic and clinical characteristics, including age, sex, disease duration, smoking status, smoking index, medical history, and concomitant medications, will be summarized descriptively and assessed for balance between groups. When appropriate, adjusted analyses will be conducted by including these variables as covariates in multivariable models to reduce the impact of potential confounding factors on treatment effect estimation. In addition, prespecified subgroup analyses stratified by sex will be performed to explore potential differences in treatment response to *Cordyceps militaris* capsules.

Safety analyses will be performed in the safety set, defined as all participants who receive at least one dose of study medication. The incidence of adverse drug reactions will be calculated based on events deemed certain, probable, and possible. Safety outcomes will be summarized by treatment group using the number and percentage of participants with any AE, adverse drug reaction or any SAE. Between-group comparisons of safety outcomes will be exploratory and conducted using Pearson’s chi-square test or Fisher’s exact test, as appropriate.

### 2.12 Quality control

To ensure study integrity and data reliability, a comprehensive researcher operations manual has been developed, detailing standardized protocols for all study procedures. Additionally, all research personnel have undergone protocol training prior to trial initiation. Following trial initiation, the main research center will conduct regular quality control visits to sub-centers based on enrollment progress. An Independent Data Monitoring Committee (IDMC) will be established for this trial, comprising at least two independent clinical experts and one statistical expert, all of whom will be unaffiliated with the study. The primary responsibilities of the IDMC include conducting independent evaluations of safety analyses, re-estimating potential sample size, and formulating evidence-based recommendations regarding protocol implementation to ensure participants’ safety and protect their rights, while maintaining strict adherence to Good Clinical Practice (GCP) guidelines throughout the trial monitoring process. The IDMC will offer guidance regarding critical matters that may emerge during trial conduct.

### 2.13 Ethical considerations

This trial adheres to the principles of GCP and the Declaration of Helsinki. The study protocol received ethical approval from the Institutional Review Board of Guangdong Provincial Hospital of Traditional Chinese Medicine (Approval No.: BF 2024-066-01). The study undergoes periodic review by the committee. Prior to enrollment, researchers will obtain written informed consent after fully explaining the study’s purpose, procedures, and potential risks. Throughout the trial, participants’ safety will be safeguarded through continuous medical supervision, with all personal health data maintained under strict confidentiality accessible solely to designated study personnel. Findings will be disseminated through peer-reviewed conferences or scientific articles while protecting participant anonymity.

### 2.14 Adverse events

All AEs are actively solicited and recorded in the CRFs, including onset time, duration, severity, seriousness, outcome, action taken, concomitant treatment, and relationship to the investigational product. Considering the herbal nature of *Cordyceps militaris* capsules and the composition of the placebo, predefined safety events of special interest include gastrointestinal symptoms (e.g., nausea, diarrhea, abdominal discomfort or distension, reduced appetite), hypersensitivity reactions (e.g., rash, pruritus, urticaria), and clinically significant hepatic or renal laboratory abnormalities.

AEs are graded using a prespecified standardized severity grading system, while the severity of acute exacerbations of COPD is classified separately according to protocol-defined COPD exacerbation criteria. Causality between AEs and the study intervention will be assessed according to the Technical Guideline for the Assessment of Causality of Adverse Events in Drug Clinical Trials issued by the China National Medical Products Administration and categorized as certain, probable, possible, unlikely, or impossible. The detailed information is shown in Supplementary Material 5.

The IDMC will periodically review safety data to assess participant safety and provide recommendations regarding the continuation, modification, or termination of the study. SAEs will receive immediate medical management and will be reported to the Principal Investigator within 24 h. Participants who experience severe or clinically significant treatment-related adverse events, or who are considered by the Investigator to be at unacceptable risk, may discontinue study medication. Whenever possible, safety follow-up should continue until the event resolves, stabilizes, or is otherwise explained.

## 3 Discussion

COPD is the third leading cause of global mortality and remains a major public health challenge because of its high prevalence, substantial disability burden and mortality rates (Collaborators, 2024). Decline in FEV_1_ has become a validated proxy for disease progression in COPD. A study has indicated an inverse relationship between the rate of lung function decline and the severity of airflow limitation in COPD, suggesting that patients with mild-to-moderate COPD may experience a relatively rapid decline in FEV_1_ during the early stage of the disease (Vestbo et al., 2011). Within this framework, multiple clinical trials have assessed whether pharmacological and non-pharmacological interventions can significantly modify the longitudinal trajectory of FEV_1_ decline. Smoking cessation reduces the risk of COPD and slows lung function deterioration, offering cost-benefit advantages over other interventions (van Eerd et al., 2016). In addition, a meta-analysis of nine randomized controlled trials demonstrated that pharmacological treatment reduced the rate of FEV_1_ decline by approximately 5.0 mL/year compared with placebo, with greater effects observed in inhaled corticosteroid-containing regimens (Celli et al., 2021). While pharmacological interventions may offer potential benefits in attenuating lung function decline, current evidence remains insufficient to conclusively establish their efficacy (Global Initiative for Chronic Obstructive Lung Disease, 2025).

COPD is characterized by chronic inflammation of the small airways and lung parenchyma, involving oxidative stress, inflammatory-cell infiltration, mucus hypersecretion, airway remodeling, peribronchiolar fibrosis, and emphysematous destruction, ultimately leading to persistent airflow limitation and progressive decline in FEV_1_ (Barnes, 2016). *Cordyceps militaris* is a medicinal fungus widely used in TCM, has been reported to improve lung function and attenuate pathological changes in COPD mice, potentially through regulation of linoleic acid metabolism and modulation of IDH1 and CYP19A1 expression (Wang et al., 2026). In addition, *Cordyceps militari*s ARA301 extract has been reported to protect against lung fibrosis and mucus deposition by reducing the secretion of pro-inflammatory cytokines induced by LPS and inhibiting the phosphorylation of p65, IκBα, and IKKα (Seong et al., 2025). Furthermore, an isoflavone glycitein isolated from *Cordyceps militaris* grown on germinated soybean extract, has demonstrated antioxidant activity and has been shown to protect NCI-H292 airway epithelial cells from EGF-induced injury by downregulating COX-2, MMP-9, and MUC5AC gene expression through inhibition of the NF-κB and p38/ERK MAPK pathways (Kim et al., 2012).

According to the HERB 2.0 database (Gao et al., 2025), *Cordyceps militaris* contains multiple bioactive constituents, including cordycepin, inosine, and adenine. Cordycepin is one of its major active constituents and is commonly used as a characteristic marker for quality control of *Cordyceps militaris* capsule preparations (Liu et al., 2023). KEGG enrichment analysis of the mapped targets highlighted the FoxO signaling pathway. The FoxO-related mapped targets included Tgfb2, Bcl6, Foxo3, Fbxo32, S1pr1, Pik3r3, and Fbxo25. Meanwhile, KEGG enrichment analysis also suggested that inosine may be associated with the FoxO signaling pathway (Gao et al., 2025). FoxO signaling regulates oxidative-stress resistance, autophagy, apoptosis, cell-cycle control, cellular senescence, and metabolic homeostasis, all of which are closely related to COPD progression and lung-function decline. FoxO3 expression has been reported to be reduced in the lungs of smokers and patients with COPD, and FOXO3 deficiency increases susceptibility to cigarette smoke-induced inflammation, airspace enlargement, and emphysema-like changes (Hwang et al., 2011). In addition, SIRT1 protects against emphysema through FoxO3-mediated reduction of premature cellular senescence (Yao et al., 2012).

Collectively, these findings suggest that *Cordyceps militaris* may exert lung-protective effects through multi-component and multi-target mechanisms. Although *Cordyceps militaris* capsules have shown efficacy in relieving related symptoms of COPD in clinic settings, its potential to modify objective pulmonary function measures requires further validation (Yu et al., 2019). Therefore, this study will evaluate the efficacy of *Cordyceps militaris* capsules in improving lung function among patients with mild-to-moderate COPD, using post-bronchodilator FEV_1_ as the primary endpoint.

Dysbiosis refers to disruption of the microbiome and has been observed in multiple compartments in patients with COPD, including the respiratory and gastrointestinal tracts. Scholars have proposed that inhaled irritants (e.g., cigarette smoke) impair pulmonary innate immunity, inducing lung microbiome dysbiosis. This triggers persistent inflammation and further weakens host defenses, forming a self-perpetuating “vicious cycle” underlying recurrent COPD exacerbations (Whiteside et al., 2021). A study has demonstrated that *Cordyceps militaris* alleviates COPD by modulating amino acid metabolism, restoring gut microbiota balance, and increasing short-chain fatty acids (Zhang et al., 2025). To elucidate the mechanism of lung-gut axis modulation in COPD therapy, we will conduct a multi-omics analysis of paired blood, sputum, and stool samples collected from mild-to-moderate COPD patients.

The primary objectives of stable COPD management include alleviating current symptoms and mitigating future disease progression risks, as outlined in the GOLD 2025 report (Global Initiative for Chronic Obstructive Lung Disease, 2025). To comprehensively evaluate treatment efficacy, this study will use multiple validated instruments: CAT to quantify disease impact on quality of life, CASA-Q for respiratory symptom burden, and mMRC dyspnea scale for breathlessness severity. Additionally, this study will assess general health status using the EQ-5D-5L and monitor exacerbation patterns through both the frequency of acute events and time to first exacerbation.

In conclusion, this study aims to evaluate the therapeutic potential of *Cordyceps militaris* capsules in preserving lung function among patients with mild-to-moderate COPD. The findings may offer evidence-based clinical insights for patient management and serve as a foundation for future research in integrative COPD therapies.
