# Supplementary material for: Evaluating Cordyceps militaris capsules on post-bronchodilator FEV1 decline in patients with COPD: a study protocol for double-blind, randomized, placebo-controlled trial
Source: Front Pharmacol. 2026 May 25;17:1775068. doi: 10.3389/fphar.2026.1775068 (PMC13243416; doi:10.3389/fphar.2026.1775068)
Supplement: Supplementary file 2 [file DataSheet5.docx]

Supplementary Material 5 Safety monitoring and causality assessment framework

# Definitions of Adverse event and Serious adverse event

Adverse events (AEs) are defined as any untoward medical occurrence after administration of the investigational product, regardless of whether it is considered related to study treatment. AEs include new symptoms or diseases, worsening of pre-existing conditions, clinically significant abnormal laboratory findings, and other medically relevant events occurring during the study period.

Serious adverse events are defined as any adverse event that results in death, is life-threatening, causes persistent or significant disability/incapacity, results in congenital anomaly/birth defect, or requires inpatient hospitalization or prolongation of existing hospitalization.

# Severity grading

The severity of each AE will be graded as follows:

(1) Mild: Easily tolerated, causing no or minimal interference with daily activities, and usually not requiring specific treatment;

(2) Moderate: Causing some interference with daily activities and usually requiring medical attention, but not placing the participant at immediate risk of permanent harm;

(3) Severe: Causing marked limitation of daily activities or significant worsening of clinical condition, and requiring intensified treatment or intervention;

# Causality assessment

Causality between the AEs and the study intervention is categorised as certain, probable, possible, unlikely or impossible, according to the Technical Guideline for the Assessment of Causality of Adverse Events in Drug Clinical Trials by China National Medical Products Administration. The relationship between AEs and the investigational product is assessed by the investigator using the prespecified causality criteria shown in **Table 1.**

**Table 1 Causality assessment of AEs in clinical trials**

| Assessment criterion | Certain | Probable | Possible | | | | Unlikely | | Impossible |
| --- | --- | --- | --- | --- | --- | --- | --- | --- | --- |
| 1. Is there a reasonable temporal relationship between drug administration and the occurrence of the adverse reaction/adverse event? | + | + | + | | | | ± | | - |
| 2. Is the reaction consistent with the known type of adverse reaction of the drug? | + | + | + | | - | | - | - | + |
| 3. Did the reaction/event resolve or disappear after drug withdrawal or dose reduction? (Dechallenge result) | + | + | + | -/? | + | -/? | - | | - |
| 4. Did the same reaction/event recur upon re-administration of the suspected drug? (Rechallenge result) | + | -/? | -/? | | | | -/? | | -/? |
| 5. Can the reaction/event be explained by the effects of concomitant medications, progression of the patient’s disease, or the influence of other treatments? | - | - | + | - | - | - | ++ | + | + |

“+” indicates yes or a positive result; “-” indicates no, a negative result, or that the result is not yet available; “±” indicates that a temporal relationship cannot be ruled out; “++” indicates that the event can be explained by other, more plausible causes; “-/?” indicates that the dechallenge or rechallenge result is negative, or that dechallenge or rechallenge has not been performed, or that dechallenge or rechallenge is not applicable.

R

S2

S1
