# Supplementary material for: Evaluating Cordyceps militaris capsules on post-bronchodilator FEV1 decline in patients with COPD: a study protocol for double-blind, randomized, placebo-controlled trial
Source: Front Pharmacol. 2026 May 25;17:1775068. doi: 10.3389/fphar.2026.1775068 (PMC13243416; doi:10.3389/fphar.2026.1775068)
Supplement: Supplementary file 3 [file DataSheet3.pdf]

Jilin Zhongsheng Pharmaceutical Co., LTD

Drug Inspection Report

Document number: R-QC-0110

Sample Name	Cordyceps militaris palcebo capsules	Size	0.25 g/capsule
Batch No.	20240501	Batch Size	200,000 capsules
License No.	/	Sample Quantity	1 inner box
Packing Size	24 capsules/pouch × 14 pouches/inner box × 6 inner boxes/carton	Sampling Date	10 May 2024
Period of Validity	4 May 2027	Report Date	17 May 2024
Inspecting Item	Complete inspection	Report No.	CP/BG-24-037
Executive Standard	Quality standard of placebo for *Cordyceps militaris* capsule(self draft); The Pharmacopoeia of the People's Republic of China 2020 Edition		

吉林省中晟制药有限公司 质检专用章

**Inspecting item**	**Standard code**	**Result**
【Description】	Pale yellow to yellowish brown powder is filled in hard gelatin capsules with a slight fishy odor and a mildly salty taste.	Pale yellowish brown powder is filled in hard gelatin capsules with a slight fishy odor and a mildly salty taste.
【Identification】		
(1) TLC Identification	Fluorescing spots corresponding in color to cordycepin reference substance should not be detected.	Fluorescing spots corresponding in color to cordycepin reference substance is not be detected.
【Inspection】		
Weight Variation	Complies with the test for weight variation	Complies with the test for weight variation
Disintegration	30 minutes	16 minutes
Water	Not more than 9.0%	5.3%
【Microbial Limit】		
Total Aerobic Microbial Count	10^4 cfu/g	Less than 10 cfu/g
Total Combined Yeasts and Moulds Count	10^2 cfu/g	Less than 10 cfu/g
Bile-Tolerant Gram-Negative Bacteria	Less than 10^2 cfu/g	Less than 10 cfu/g
Escherichia coli	Not to be detected (1 g)	Not detected (1 g)
Salmonella	Not to be detected (10 g)	Not detected (10 g)
【Gelatin hollow capsules】	Batch No. 2305238	
Chromium	Not exceed two parts per million.	1×10^{-6}

Conclusion: The product was tested in accordance with Quality standard of placebo for *Cordyceps militaris* capsule(self draft) and The Pharmacopoeia of the People's Republic of China 2020 Edition. The results complied with the specifications.

有限公司 用章

Prepared by: 丁雷 Reviewed by: 李金香 Approved by: 黄利

吉林省中晟制药有限公司

药品检验报告书

文件编号：R-QC-0110

检品名称	蛹虫草菌粉胶囊安慰剂	规　格	每粒装 0.25g
产品批号	20240501	批　量	20 万粒
批准文号	/	取样数量	1 盒
包装规格	24 粒/袋×14 袋/中盒×6 中盒/箱	取样日期	2024 年 05 月 10 日
有效期至	2027 年 05 月 04 日	报告日期	2024 年 05 月 17 日
检验项目	全检	报告书编号	CP/BG-24-037
检验依据	《蛹虫草菌粉胶囊安慰剂质量标准（自拟）》、《中国药典》2020 年版四部		

检验项目	标准规定	检验结果
【性状】	本品为胶囊剂，内容物为浅黄色至黄棕色粉末；气微腥，味微咸。	本品为胶囊剂，内容物为浅黄棕色粉末；气微腥，味微咸。
【鉴别】		
（1）薄层鉴别	不应检出与虫草素对照品相应颜色的荧光斑点。	未检出与虫草素对照品相应颜色的荧光斑点。
【检查】		
装量差异	应符合规定	符合规定
崩解时限	应在 30 分钟内全部崩解	16 分钟
水分	不得过 9.0%	5.3%
【微生物限度】		
需氧菌总数	10^4cfu/g	小于 10cfu/g
霉菌及酵母菌总数	10^2cfu/g	小于 10cfu/g
耐胆盐革兰阴性菌	应小于 10^2cfu/g	小于 10cfu/g
大肠埃希菌	应不得检出（1g）	未检出（1g）
沙门菌	应不得检出（10g）	未检出（10g）
【所用明胶空心胶囊】	批号 2305238	
铬	应不得过百万分之二	1×10^{-6}

-----以下空白-----

结论：本品按《蛹虫草菌粉胶囊安慰剂质量标准（自拟）》、《中国药典》2020 年版四部检验，结果符合规定。

起草人：丁雪　　　　审核人：李金香　　　　负责人：黄利

01 版

启用日期：2019 年 11 月 25 日
