# Supplementary material for: Evaluating Cordyceps militaris capsules on post-bronchodilator FEV1 decline in patients with COPD: a study protocol for double-blind, randomized, placebo-controlled trial
Source: Front Pharmacol. 2026 May 25;17:1775068. doi: 10.3389/fphar.2026.1775068 (PMC13243416; doi:10.3389/fphar.2026.1775068)
Supplement: Supplementary file 4 [file DataSheet2.docx]

Supplementary Material 2 The composition analysis of Cordyceps militaris capsules and the fingerprint of quality control

# Materials and methods

## Cordycepin

Determination by high-performance liquid chromatography (HPLC).

**Chromatographic conditions and system suitability test:** Octadecyl silane bonded silica gel is used as filler. Methanol-phosphate buffer solution (pH 6.5)(15:85) is used as the mobile phase. The detection wavelength is 260nm. The number of theoretical plates shall not be less than 3000 according to the peak of cordycepin.

**Preparation of reference solution:** Take an appropriate amount of cordycepin as the reference substance and weigh accurately. Add methanol to make a solution containing 30µg per 1ml.

**Preparation of test solution:** Take the capsule contents from the weight variation test, grind into a fine powder, and accurately weigh approximately 0.25 g. Add 25 mL of methanol, and weigh it. Heat under reflux for 2 hours, then cool to room temperature. Weigh it again and add methanol to compensate for the weight loss. Mix well and filter through a 0.45 μm microporous membrane.

**Determination method:** Accurately absorb the control solution and the test solution 10 μl respectively. Inject them into the liquid chromatograph for determination.

**Calculated as dry product:** The content of cordycepin(C_10_H_13_N_5_O_3_) per 1 granule shall not be less than 0.125mg.

## Mannitol

Determination by gas chromatography(GC).

**Chromatographic conditions and system suitability test:** The chromatographic column is carried out on HP-5MS with 5% diphenyl and 95% dimethyl polysiloxane as stationary phase. A capillary column (30 m × 250 μm × 0.25 μm) is used. The column temperature is programmed as follows: initial temperature at 100 °C, increased at 20 °C/min to 250 °C (held for 4 min), then increased at 20 °C/min to 280 °C (held for 4 min). The carrier gas is nitrogen at a flow rate of 0.8 mL/min. The injection is performed in splitless mode with the injector temperature at 200 °C and the detector (FID) temperature at 300 °C. The number of theoretical plates shall not be less than 1500 according to the peak of mannitol hexaacetate.

**Preparation of reference solution:**Accurately weigh 5 mg of the mannitol reference substance (dried to constant weight at 105 °C), place it in a 20 mL headspace vial, and add 5 mL of anhydrous pyridine (previously dehydrated with anhydrous sodium sulfate) and 10 mL of acetic anhydride. Mix well, seal the vial, and heat in an oven at 90 °C for 1 hour. Remove the vial from the oven and allow it to cool to room temperature. Open the vial and transfer the contents to a centrifuge tube. Centrifuge the mixture and collect the supernatant to obtain the product.

**Preparation of test solution:** Accurately weigh 75 mg of the capsule contents(dried overnight placement in phosphorus pentoxide dryer), place it in a 20 mL headspace vial, and add 2.5 mL of anhydrous pyridine (previously dehydrated with anhydrous sodium sulfate) and 5 mL of acetic anhydride. Mix well, seal the vial, and heat in an oven at 90 °C for 1 hour. Remove the vial from the oven and allow it to cool to room temperature. Open the vial and transfer the contents to a centrifuge tube. Centrifuge the mixture and collect the supernatant to obtain the product.

**Determination method:** Accurately absorb the control solution and the test solution 0.6 μl respectively. Inject them into the gas chromatograph for determination.

**Calculated as dry product:** The content of mannitol（C_6_H_14_O_6_） per 1 granule shall not be less than 3.7mg.

# Result

The HPLC fingerprint of seventeen batches of *Cordyceps militaris* capsules and eight reference compounds has been studied (Figure 1). The composition and content of *Cordyceps militaris* capsules is shown in Table 1. The GC fingerprint of ten batches of *Cordyceps militaris* capsules and mannitol reference has been studied (Figure 2). The mannitol content of *Cordyceps militaris* capsules is shown in Table 2.

R4

R3

R2

S17

S16

S15

S14

S13

S11

S12

S10

S9

S8

S7

S6

S4

S5

S3

S2

R1

S1

**Supplementary Figure 1.** HPLC fingerprint profiles of *Cordyceps militaris* capsule samples (S1-S17) and reference samples (R1-R4). 1.Uracil; 2. Cytidine; 3. Hypoxanthine; 4. Uridine; 5. Adenine; 6. Guanosine; 7. Adenosine; 8. Cordycepin; R1. Mixed control (containing Cytidine, Uridine, Guanosine, Adenosine and Cordycepin); R2. Xanthine control; R3. Uracil control; R4. Adenine control

Table 1 The composition and content of Cordyceps militaris capsules/%

| **Batch** | **Uracil** | **Cytidine** | **Hypoxanthine** | **Uridine** | **Adenine** | **Guanosine** | **Adenosine** | **Cordycepin** |
| --- | --- | --- | --- | --- | --- | --- | --- | --- |
| S1 | 0.009 | 0.070 | 0.009 | 0.144 | 0.013 | 0.07 | 0.086 | 0.076 |
| S2 | 0.005 | 0.105 | 0.006 | 0.109 | 0.009 | 0.089 | 0.111 | 0.113 |
| S3 | 0.009 | 0.1210 | 0.008 | 0.205 | 0.011 | 0.112 | 0.144 | 0.123 |
| S4 | 0.009 | 0.1170 | 0.008 | 0.198 | 0.011 | 0.107 | 0.139 | 0.117 |
| S5 | 0.006 | 0.124 | 0.006 | 0.201 | 0.008 | 0.121 | 0.162 | 0.117 |
| S6 | 0.004 | 0.126 | 0.005 | 0.215 | 0.007 | 0.13 | 0.177 | 0.122 |
| S7 | 0.004 | 0.127 | 0.000 | 0.214 | 0.006 | 0.128 | 0.176 | 0.120 |
| S8 | 0.008 | 0.110 | 0.008 | 0.207 | 0.012 | 0.11 | 0.151 | 0.122 |
| S9 | 0.010 | 0.094 | 0.011 | 0.225 | 0.017 | 0.095 | 0.133 | 0.135 |
| S10 | 0.010 | 0.093 | 0.011 | 0.224 | 0.017 | 0.093 | 0.133 | 0.136 |
| S11 | 0.007 | 0.094 | 0.007 | 0.152 | 0.01 | 0.106 | 0.157 | 0.144 |
| S12 | 0.007 | 0.098 | 0.007 | 0.147 | 0.01 | 0.11 | 0.156 | 0.137 |
| S13 | 0.007 | 0.101 | 0.007 | 0.154 | 0.01 | 0.113 | 0.159 | 0.137 |
| S14 | 0.007 | 0.106 | 0.007 | 0.160 | 0.010 | 0.117 | 0.165 | 0.138 |
| S15 | 0.013 | 0.080 | 0.010 | 0.157 | 0.016 | 0.091 | 0.144 | 0.137 |
| S16 | 0.013 | 0.079 | 0.010 | 0.152 | 0.016 | 0.088 | 0.138 | 0.133 |
| S17 | 0.010 | 0.107 | 0.007 | 0.176 | 0.012 | 0.111 | 0.153 | 0.13 |
| Average content | 0.008 | 0.103 | 0.007 | 0.179 | 0.011 | 0.105 | 0.146 | 0.126 |
| coefficient of variation | 0.328 | 0.165 | 0.348 | 0.193 | 0.294 | 0.149 | 0.155 | 0.126 |

R

S10

S7

S6

S9

S8

S3

S5

S2

S4

S1

**Supplementary Figure 1.** GC fingerprint profiles of *Cordyceps militaris* capsule samples (S1-S10) and mannitol control (R). 1. Mannitol; R. Mannitol control

**Table 2 The content of Mannitol in samples of *Cordyceps militaris* capsules**

| **Batch** | S1 | S2 | S3 | S4 | S5 | S6 | S7 | S8 | S9 | S10 |
| --- | --- | --- | --- | --- | --- | --- | --- | --- | --- | --- |
| **Content（mg/g）** | 8.8 | 8.7 | 9.2 | 9.3 | 9.1 | 8.8 | 8.6 | 8.9 | 9.1 | 9.3 |
