# Supplementary material for: Evaluating Cordyceps militaris capsules on post-bronchodilator FEV1 decline in patients with COPD: a study protocol for double-blind, randomized, placebo-controlled trial
Source: Front Pharmacol. 2026 May 25;17:1775068. doi: 10.3389/fphar.2026.1775068 (PMC13243416; doi:10.3389/fphar.2026.1775068)
Supplement: Supplementary file 5 [file DataSheet1.docx]

Supplementary Material 1 The preparation of Cordyceps militaris and placebo capsules

# *Cordyceps militaris* capsules

## The composition of *Cordyceps militaris* capsules（each batch of 1,000 capsules）

| Name | Dosage | Grade | Manufacturer |
| --- | --- | --- | --- |
| *Cordyceps militaris* | 208 g | Pharmaceutical | Jilin Zhongsheng Pharmaceutical Co., LTD |
| Corn starch | 42 g | Pharmaceutical | Liaoning Dongyuan Pharmaceutical Co., LTD |

## The preparation of *Cordyceps militaris* capsules（each batch of 1,000 capsules）

*Cordyceps militaris* strain were inoculated onto oak silkworm pupae and cultured at 15-22℃ for approximately 45 days. After growing stroma, the material was harvested, dried at 60°C and ground into a fine powder. For each batch of 1,000 capsules, 208 g of *Cordyceps militaris* powder and 42 g of dried corn starch were homogenized in a dual-base hopper mixer (BDV1500, Chongqing Hanweidi Technology Co., LTD.) for 30 minutes at 8 rpm. The blended powder were then encapsulated using an automatic capsule filler (NJP-3500B, Beijing Hanlin Hangyu Technology Development Inc.), with fill weight controlled within 0.25g±7.5% (0.232–0.268g). Finally, the capsules were polished with an automatic capsule polishing machine (JNFP-2, Beijing Hanlin Hangyu Technology Development Inc.) to obtain the finished product.

# *Cordyceps militaris* placebo capsules

## The composition of *Cordyceps militaris* placebo capsules（each batch of 1,000 capsules）

| Name | Dosage | Grade | Manufacturer |
| --- | --- | --- | --- |
| Dextrin | 208 g | Pharmaceutical | Liaoning Dongyuan Pharmaceutical Co., LTD |
| Corn starch | 17.25 g | Pharmaceutical | Liaoning Dongyuan Pharmaceutical Co., LTD |
| Caramel | 12.2 g | Food | Quanwei Xian Seasoning Flagship Store |
| Tartrazine | 0.03 g | Food | Shanghai Dyestuffs Research Institute CO., LTD |
| Lentinus Edodes (Shiitake) Powder | 22.5 g | Food | Hanzhong Qinnongyuan Ecological Development Co., LTD. |
| Dried Flatfish Powder | 2.25 g | Food | Guangdong Niannian Youyu Food Co., LTD. |

## The preparation of *Cordyceps militaris* placebo capsules（each batch of 1,000 capsules）

The wetting agent was prepared by dissolving tartrazine and caramel in purified water (Beijing InnoGreen Technology Co., LTD.) and stirring uniformly. Dextrin and corn starch were mixed in a hopper mixer (BCV300, Chongqing Hanweidi Technology Co., LTD.) for 30 minutes at 10 rpm. The pre-mixed powder was transferred to a trough mixer (CH200A, Jiangsu Pioneer Engineering Drying Co., Ltd.), where the wetting agent is added and stirred uniformly for 5 minutes. A 20-mesh screen is installed on a shaking granulator (YK-160C, Jiangsu Pioneer Engineering Drying Co., LTD.). The prepared soft material is fed into the hopper of the shaking granulator and dried in a hot air circulation oven (CT-C-Ⅱ, Nanjing Baowei Drying Machinery Factory) using stainless steel trays at 60±5°C for 3-4 hours. The dried material is then processed through a water-cooled dust extraction and crushing unit (WSX-320, Nanjing Baowei Drying Machinery Factory) on a 100-mesh sieve. Finally, the crushed material, shiitake powder, and Dadiyu powder are mixed in a hopper mixer (BCV300, Chongqing Hanweidi Technology Co., LTD.) for 30 minutes at 10 rpm. The qualified preparation mixed powder was filled with the automatic capsule filling machine (NJP-3500B, Beijing Hanlin Hangyu Technology Development Co., LTD.), and the filling difference was controlled at 0.25g+7.5% (0.232~0.268g). The capsule was polished with the capsule sorting and polishing machine (JNFP-2, Beijing Hanlin Hangyu Technology Development Co., Ltd.), and the capsule was obtained.

# The sample of *Cordyceps militaris* and placebo capsules


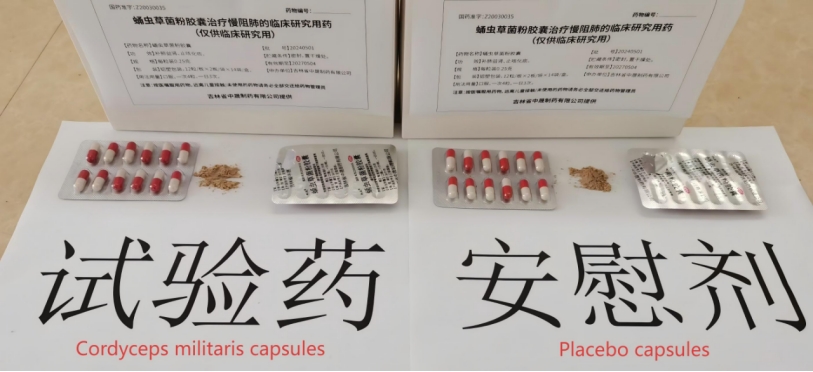


**Supplementary Figure 1.** *Cordyceps militaris* and placebo capsules.
